# Minimally Invasive Surgical Therapies for Atrial Fibrillation

**DOI:** 10.5402/2012/606324

**Published:** 2012-05-16

**Authors:** Yoshitsugu Nakamura, Bob Kiaii, Michael W. A. Chu

**Affiliations:** ^1^Division of Cardiac Surgery, Department of Surgery, Lawson Health Research Institute, University of Western Ontario, London, ON, Canada; ^2^Division of Cardiac Surgery, Department of Surgery, London Health Sciences Centre, B6-106 University Hospital, 339 Windermere Road, London, ON, Canada N6A 5A5

## Abstract

Atrial fibrillation is the most common sustained arrhythmia and is associated with significant risks of thromboembolism, stroke, congestive heart failure, and death. There have been major advances in the management of atrial fibrillation including pharmacologic therapies, antithrombotic therapies, and ablation techniques. Surgery for atrial fibrillation, including both concomitant and stand-alone interventions, is an effective therapy to restore sinus rhythm. Minimally invasive surgical ablation is an emerging field that aims for the superior results of the traditional Cox-Maze procedure through a less invasive operation with lower morbidity, quicker recovery, and improved patient satisfaction. These novel techniques utilize endoscopic or minithoracotomy approaches with various energy sources to achieve electrical isolation of the pulmonary veins in addition to other ablation lines. We review advancements in minimally invasive techniques for atrial fibrillation surgery, including management of the left atrial appendage.

## 1. Introduction

Atrial fibrillation (AF) is the most common cardiac arrhythmia, affecting over 2 million people in the United States [1]. The lifetime risks for developing AF are 1 in 6, even in those without previous cardiac disease and as high as 1 in 4 in those individuals older than 40 years [2]. It is associated with nearly a five-fold increased risk of stroke and over two-fold increase risk of death [3]. There have been many advances in the management of AF including pharmacological therapies, antithrombotic therapies, and ablation techniques. Concurrently, minimally invasive cardiac surgery techniques have emerged to treat selected patients with AF, refractory to medical therapy, with surgical ablation and left atrial appendage (LAA) resection through much smaller, sternal sparing incisions. These innovative techniques focus on pulmonary vein isolation, ablation of the left atrial isthmus and right atrial isthmus in addition to other ablation lines and can be performed as either a stand-alone operation or concomitant with another minimally invasive cardiac operation. The goals of minimally invasive AF surgeries are to achieve the same success in restoring sinus rhythm as the conventional Cox-Maze procedure with a more cosmetically appealing incision, quicker recovery, and improved patient, satisfaction.

## 2. History of AF Surgery

 It has been recognized that in patients with chronic AF, pharmacological rhythm control is ineffective in half of patients, and electrical cardioversion has high recurrence rates [4, 5]. As a result, surgical ablation techniques were developed over the past 30 years to address medically refractory patients with few other options. In 1980, Williams and coworkers developed an arrhythmia procedure which isolated the left atrium electrically from the remainder of the heart to confine AF to the left atrium [6]. In 1990, Guiraudon and coworkers described a more aggressive technique, the Corridor procedure [7], which created an isolated strip of muscle connecting the sinus node to atrioventricular node. These two procedures had some effect to restore sinus rhythm; however, the shortcomings of these two procedures were continued fibrillation of both atria with persistent risk of thromboembolism and lack of atrial transport function.

**Figure 1:**
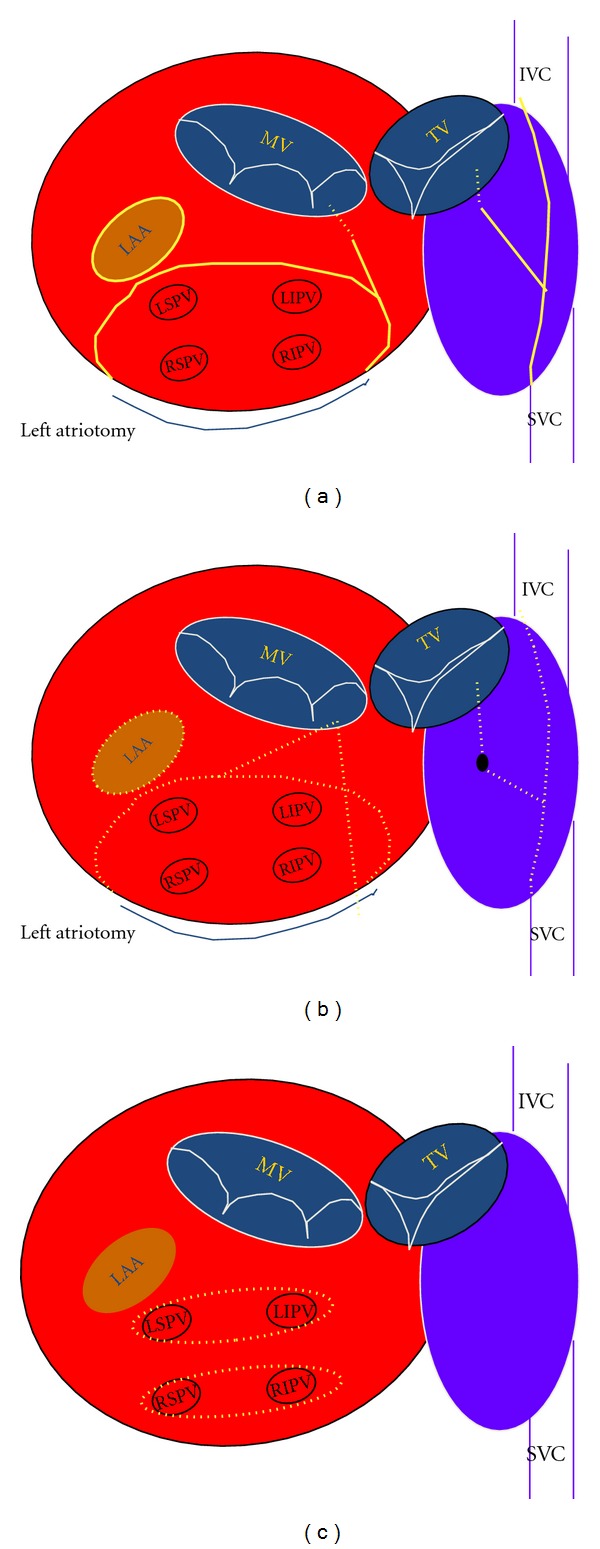
Lesion sets. (a): traditional Cox-maze III operation, (b): Cryomaze procedure, (c): commonly employed epicardial radiofrequency ablation. The solid lines indicate the “cut and sew” incisions, and the broken lines indicate the ablation lines. MV: mitral valve, TV: tricuspid valve, IVC: inferior vena cava, SVC: superior vena cava, LAA: left atrial appendage, LSPV: left superior pulmonary vein, LIPV: left inferior pulmonary vein, RSPV: right superior pulmonary vein, and RIPV: right inferior pulmonary vein.

In 1991, Cox and coworkers reported the Maze procedure based on experimental and early clinical evidence [8]. The Maze operation consisted of several atrial disconnections (“cut and sew”) to create lines of electrical block for pulmonary vein isolation and interruption of all potential macroreentrant circuits. The Maze procedure was modified twice to improve the left atrial transport function and to simplify the operation (Cox-Maze II and III). In 1996, they reported the series of 118 patients undergoing the Cox-Maze III procedure (Figure 1(a)), which achieved an operative mortality of 2%, freedom from AF was 93% in the mean followup of 8.5 years [9].

Although the Cox-maze III procedure was effective, it was not widely adopted because of its overall complexity and invasiveness. In late 1990s, radiofrequency, cryoablation, and other energy sources were developed to simplify the “cut and sew” technique. In 1998, Haïssaguerre and coworkers reported a landmark study that suggested that AF originated from ectopic beats within the pulmonary veins [10] which further confirmed the rationale behind the maze procedure. Since then, AF surgery continues to focus on left atrial isolation but has been progressing towards more complete lesion sets with minimally invasive techniques to allow optimal treatment of AF through less invasive methods. Patients continue to demand more effective and less invasive therapies, particularly in cases of lone atrial fibrillation.

## 3. Development of Minimally Invasive AF Surgery

 Minimally invasive techniques were introduced to cardiac surgery in the mid-1990s, with the blending of reduced incision sizes, sternal-sparing minithoracotomy approaches, endoscopic visualization, and modified cardiopulmonary bypass techniques to enable safe and effective, less invasive valve surgery [11–13]. Over the past decade, alternative energy sources have been developed for arrhythmia surgery that have facilitated a paradigm shift towards minimally invasive AF ablation surgery. As we outline further ahead, there are many different general approaches, incisions, energy sources, lesion sets, and perfusion strategies that have been described as “minimally invasive.” There remains no clear consensus on what determines an operation to be “minimally invasive,” as most definitions focus on the potential improved outcomes, rather than the procedural details themselves. In general with regards to minimally invasive AF operations, surgical invasiveness and operative efficacy may be somewhat inversely proportional, where the ideal minimally invasive AF operation offers a patient the least “invasiveness” with maximal efficacy which will certainly vary based upon AF characteristics, duration of AF, left atrial size, and other patient related factors. In this paper, we define minimally invasive AF surgery as those approaches that employ smaller, sternal-sparing incisions using less invasive alternative energy sources with or without cardiopulmonary bypass.

## 4. Alternative Energy Sources

Ablation techniques are dependent on the ability to create electrical conduction blocks within the heart to interrupt all potential macroreentrant circuits. The classic method in surgery to create conduction block has been the “cut and sew” technique (Figure 1(a)). It has been the most reliable method for creating transmural lesions that block atrial conduction; however, is also associated with significant risks of hemorrhage and prolonged aortic cross clamp times.

Over the past decade, there has been significant progress in the development of alternative ablative devices with different energy sources that are safer alternatives to the “cut and sew” technique. In order to creation transmural atrial lesions to block electrical conduction, tissue must be subjected to either hypothermic (<−60°C) or hyperthermic (>50°C) damage. Hypothermic energy is delivered by cryothermy, while hyperthermic energies include radiofrequency (RF), microwave, ultrasound, and laser. The ideal energy source would create well-demarcated, transmural lesions through either epicardial or endocardial application with a quick, cost-effective device that can be utilized minimally invasively and does not encourage endocardial thrombus formation. We will review commonly utilized energy sources and highlight specific advantages and disadvantages.

### 4.1. Cryothermy

Cryothermy was the first energy source to be used in arrhythmia surgery and was one of the most common alternative and additive energy sources to the “cut and sew” method. Cryothermy uses argon which achieves tissue temperatures of −150°C or nitrous oxide which achieves temperatures of −60°C. The main advantages of cryothermy are the ability to create long lesion lines with modern flexible probes and the ability to mimic the entire lesion set of the Cox-maze III operation [14, 15]. In fact, the original Cox-maze procedure included cryothermy of the left atrial isthmus and a tissue gap under the left atrial appendage [8, 9].

Cryothermy has several other potential advantages including less risk of endocardial thrombus [16, 17] and lower risk of collateral damage to surrounding structures such as mitral leaflet tissue, the circumflex coronary artery, the coronary sinus, or the esophagus [17–19]. The main disadvantage of cryothermy is its poor efficacy during off pump or beating heart procedures from an epicardial approach because the warming effect of endocardial blood flow [14, 15, 19, 20]. Another disadvantage is the ablative time requirement of 60–120 seconds for freezing and the subsequent time for thawing to reposition the probe.

### 4.2. Radiofrequency (RF)

RF is a simple and effective energy source that has been widely used in catheter-based ablation. The application of RF energy increases the tissue temperature to 50–55°C with consequent coagulation and permanent destruction of cell structure and collagen [17, 21]. Available RF surgical devices include irrigated or dry application, and unipolar or bipolar systems. The addition of irrigation is believed to allow for the creation of deeper and theoretically more complete lesion lines.

Unipolar devices are usually flexible probes that allow lesion creation in flat and angulated areas; however, lesion gaps can be more common. Bipolar RF devices commonly have a “C clamp” shape, where the energy source is delivered between the electrodes mounted between both jaws of the clamp. Advantages of bipolar RF over unipolar RF are ability to make quick lesion sets (approximately 10 seconds), directional energy delivery, with theoretically less risk of collateral tissue damage, and determination of transmurality with impedance monitoring. Bipolar RF devices have been widely accepted as a safe and effective ablation for pulmonary vein isolation in minimally invasive AF surgery [21].

The main disadvantage of RF is the risk of intracavity thrombus formation. In fact, RF is considered the most thrombogenic energy source, with reports of embolic complications with coil-tipped catheters [22]. Collateral tissue damage with esophageal perforation and left circumflex artery injury has been reported as well [17, 23, 24]. Careful placement of bipolar RF devices, rather than unipolar, should help to minimize these risks. Bipolar RF devices are generally not used to create the connecting lesion to the mitral annulus because injurious risks to the circumflex artery and mitral leaflet tissue.

### 4.3. Microwave

Microwave generates heat by causing vibration and rotation of water molecules. These devices consist of a generator system and an antenna mounted on a shielded shaft to deliver microwaves to the site of ablation [17, 25–27]. The duration of application is usually 60–120 seconds. Wisser and coworkers found similar results when comparing microwave and RF ablations when performing the Cox-maze III procedure [25]. At the 12 months, freedom from AF was 81% and 80% in the microwave and RF groups, respectively. Topkara and coworkers reported similar findings in performing a lesion set similar to the Cox-maze III, with freedom from AF of 66% and 71% in microwave and RF ablation, respectively [26].

Advantages of microwave technology are lower risks of thromboembolism, minimal char formation, and the creation of a well-demarcated area of the thermal injury. Microwave is not only capable of producing transmural lesions when applied to the epicardial surface but also may avoid collateral damage because excess energy is absorbed by blood elements. Furthermore, the microwave probe (Flex-10, AFX Inc, Fremont, CA, USA) is flexible and long, making it amenable to minimally invasive techniques. Microwave was the first energy source to be used in thoracoscopic AF surgery [27]. Although complications are rare, the epicardial connecting lesion to mitral valve annulus is avoided because of concerns of circumflex coronary artery intimal damage [28].

### 4.4. Laser

Laser ablation uses high-energy optical waves to create a narrow, deep, and well-demarcated lesion with minimal lateral expansion from both endocardial and epicaridal approach [17, 29]. Animal studies have demonstrated that laser ablation is able to produce rapid, histologically transmural lesions capable of electrically isolating the atrium. The tissue is ablated by direct heating to relatively low temperatures (50°C) and also by mechanical damage from cellular lysis caused by shock waves. Hamman and Theologes reported left-sided lesion sets with laser ablation in patients with paroxysmal AF and biatrial lesion sets in those with persistent or permanent AF with an overall 76% freedom from AF up to 18 months [29]. 

### 4.5. Ultrasound

High-intensity focused ultrasound (HIFU) uses acoustic energy to create a hyperthermic transmural lesions. Its theoretical advantage is its proposed ability to create deeper and hypothetically more complete lesions than other energy sources. HIFU is capable of producing transmural lesions from an epicardial approach, in short period of time (less than 2 seconds) [17, 30].

Ninet and coworkers have reported the initial clinical experience with HIFU in a multicenter trial [30]. An epicardial ablation was performed on the beating heart before undergoing the concomitant procedure in 103 patients with either permanent (74%), paroxysmal (21%), or persistent (5%) AF. All patients underwent pulmonary vein isolation (box lesion), and an additional mitral line was created epicardially in 34%. At the 6-month period, freedom from AF was 85% in the entire study group. Unfortunately, a case of fatal atrioesophageal fistula was reported at 31 days postoperatively [31]. Because of issues of limited experience and difficulty using the bulky device, HIFU has not gained widespread acceptance for treatment of atrial fibrillation.

## 5. Minimally Invasive Approaches

Haissaguerre's work with ectopic beats within the pulmonary veins has spurred the growth of catheter-based ablation techniques for AF [10]. At present, it remains the least invasive treatment for AF; however, catheter based approaches often require more than one procedure and have considerable risks of recurrent AF, especially in patients with persistent AF [32–34]. Miyazaki et al. have reported that during 30 months of followup after the initial ablation, 59% of patients with persistent AF were free of atrial tachyarrhythmia [33]. Pokushalov et al. have reported that 12-month freedom from AF after catheter ablation for persistent AF was 48% after 1 ablation procedure and 64% after 1 or more procedures [34]. These findings continue to encourage the growth of surgical ablation techniques to develop less invasive but more effective alternatives. Arrhythmia surgery has also faced the same less invasive trends as other conventional cardiac operations. Sternal-sparing, direct vision minithoracotomy, endoscopic and robotic approaches have been developed to treat both lone AF and more commonly as concomitant therapy with mitral valve disease. These minimally invasive techniques seek to achieve the same very high standards of the Cox-maze operation with lower risks, less morbidity, quicker recovery, and improved patient satisfaction. The development of minimally invasive AF surgery has been based on two factors: more simplified and targeted lesion sets and the introduction of the previously mentioned specialized ablation devices to replace the traditional “cut and sew” maze procedure. Epicardial ablative devices have allowed for the evolution of off-pump, thoracoscopic approaches. The hallmark lesion set of minimally invasive AF surgery still remains pulmonary vein isolation. However, recent advances in thoracoscopic approaches have allowed for the expansion of more complex lesion sets to improve patient results. Right-sided lesions, autonomic denervation, left atrial appendectomy in addition to concomitant valvular surgery can all be performed simultaneously, further enhancing the strength and utility of these minimally invasive approaches.

## 6. Minimally Invasive Cryomaze Procedure

The biatrial Cryomaze procedure utilizes cryotherapy to replicate all of the original Cox-maze III lesion sets, without the same invasiveness and bleeding risks (Figure 1(b)) [35–38]. Results with the Cryomaze procedure have demonstrated efficacy for both paroxysmal and permanent AF [15, 36, 37]. In fact in patients with permanent AF, the Cryomaze procedure has provided comparable maintenance of sinus rhythm with less cross-clamp time and bleeding compared to the traditional “cut and sew” maze procedure [38]. Freedom from AF after the Cryomaze procedure is reported to be 60–80% at postoperative 3 years [15, 36].

**Figure 2:**
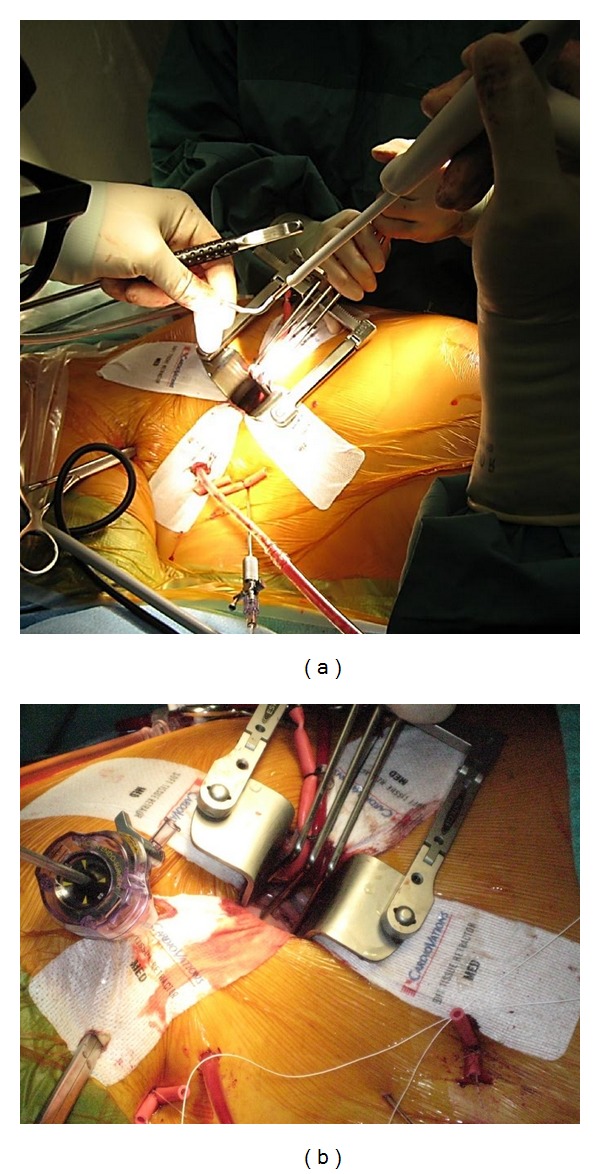
(a) intraoperative view of minimally invasive concomitant mitral valve repair and Cryomaze procedure. The argon-based flexible, linear ablation probe is applied through a 3 cm right minithoracotomy. (b) intraoperative view of minimally invasive, videoscopic Cryomaze setup.

The Cryomaze can be performed endoscopically, robotically or through a 3-4 cm right minithoracotomy using cardiopulmonary bypass with femoro-femoral cannulations and right jugular venous cannulation (Figures 2(a) and 2(b)) [35, 37]. The CryoCath ablation system (Medtronic Inc., MN, USA) uses argon to cool tissue to as low as −160°C. The system is well suited to a minimally invasive technique because it is a flexible, linear probe available in lengths of 6–10 cm. The minimally invasive Cryomaze procedure has also demonstrated excellent results in the lone AF population [37]. A total of 41 patients with lone AF underwent a minimally invasive, biatrial Cryomaze with no deaths or strokes. At discharge and 6 weeks postoperatively, 36 (87.8%) and 37 (90.2%) patients were in sinus rhythm, respectively. At later followup, sinus rhythm was present in 93% (38/41) at 3 months, 87% (34/39) at 6 months, and 87% (20/23) at one year [37].

**Figure 3:**
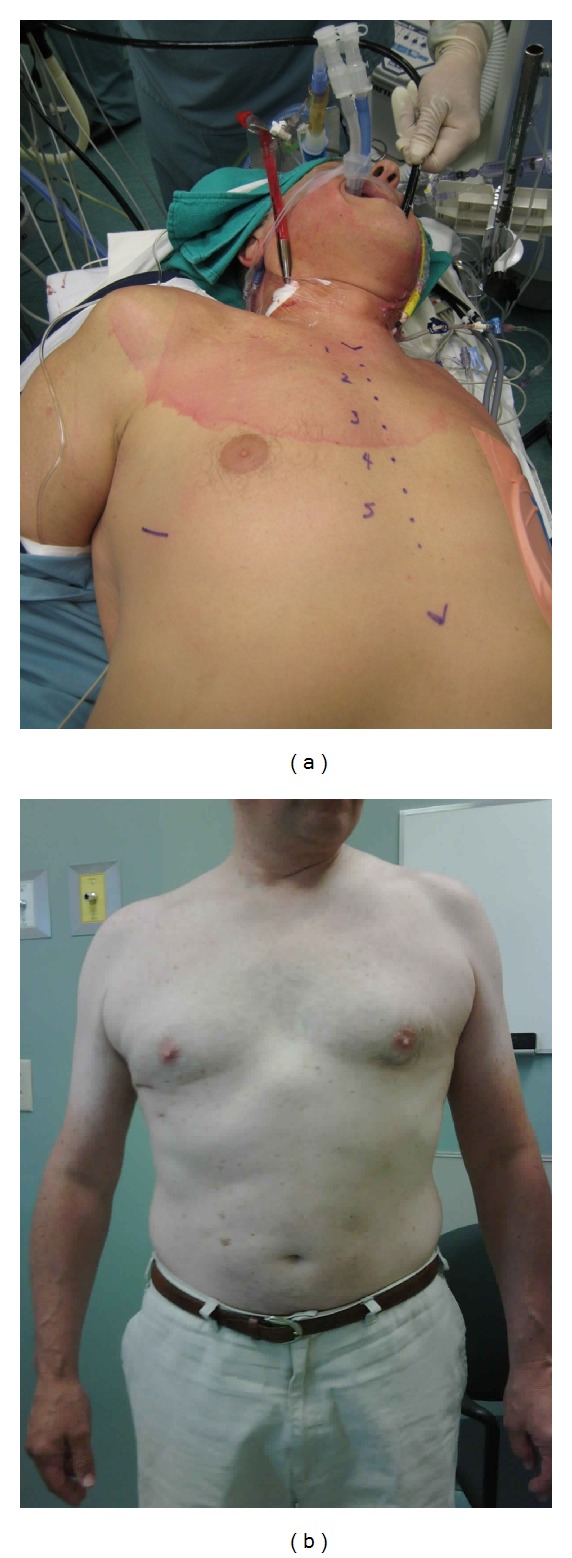
Operative setup for minimally invasive biatrial Cryomaze procedure with concomitant mitral valve repair. (a): intraoperative patient positioning, (b): 2 months postoperative result.

The biatrial Cryomaze procedure is our preferred method of surgical treatment for atrial fibrillation. We treat patients with symptomatic paroxysmal or permanent AF with a duration <10 years, left atrial size <6.5 cm, who have strong motivations to be anticoagulation free. Most patients that we treat also have concomitant mitral valve disease that requires surgical intervention. We employ videoscopic assistance to perform the operation through a 3-4 cm right minithoracotomy with peripheral cannulation (Figures 2 and 3). Following the ablation, prior to the mitral valve repair, we generally perform a left atrial appendectomy by invaginating, excising, and oversewing the stump of the left atrial appendage from within the left atrium. We have experienced good success rates with freedom from recurrent atrial fibrillation >90% and 80% in patients with paroxysmal and persistent AF, respectively.

## 7. Minimally Invasive Radiofrequency Maze Procedure

Minimally invasive AF surgery with RF ablation techniques can be performed with unipolar or bipolar devices. Most approaches have focused on epicardial pulmonary vein ablation alone with “clamp” bipolar devices around the pulmonary veins (Figure 1(c)); however, more complex lesion sets have also been added with unipolar RF or adjunct cryoablation.

In 2002, Damiano and Gaynor described the Cox-maze IV procedure using bipolar RF through standard sternotomy and cardiopulmonary bypass [39]. In this procedure, a bipolar RF clamp was used to create conduction block in most of the atrial lines; however, linear cryoablation was still required for mitral and tricuspid valve annuli in the vast majority cases. In 2010, the same group described the minimally invasive Cox-maze IV procedure through a right mini-thoracotomy with cardiopulmonary bypass, peripheral cannulation, and aortic cross clamping [40]. This procedure mimics the open Cox-maze IV lesion set, performed through direct vision and minithoracotomy. The freedom from AF in 22 patients was 94% and 81% without antiarrhythmic medications at 6 and 12 months, respectively. In 2011, Damiano and coworkers reported series of 282 patients undergoing Cox-maze IV procedure since 2002 (42% paroxysmal and 58% persistent AF) [41]. The freedom from atrial fibrillation was 93% and 89% at 6 and 12 months, respectively. The freedom from both atrial fibrillation and antiarrhythmic drugs was 79% and 78% at 6 and 12 months, respectively.

In 2004, Sie and coworkers reported an RF-based maze procedure with similar lesion set to Cox-maze IV as an adjunct to open heart surgery under cardiopulmonary bypass and cross clamp [42, 43]. The difference from Cox-maze IV was that they created only endocardial linear ablation lines using unipolar irrigated RF. They reported 44-month follow-up results of their RF-based maze in 258 patients with structural heart disease and permanent AF [44]. Sustained sinus rhythm, including atrial rhythm or an atrial-based paced rhythm was present in 69% of patients at 1 year, in 56% at 3 years, and in 52% at 5 years.

Kiser and coworkers described a totally extracardiac maze (Ex-Maze) procedure which closely mimics the Cox-maze III lesion set by using vacuum-integrated radiofrequency ablation device on the beating heart [45]. The Ex-Maze procedure was performed during 44 concomitant cardiac procedures for patients with AF; of these, 82% of the patients had persistent or permanent AF. Freedom from AF was 66% and 79% at postoperative 1 month and 3 month, respectively.

## 8. Bilateral Video-Assisted Thoracoscopic AF Surgery

In 2005, Wolf and coworkers reported their initial experience with bilateral video-assisted thoracoscopic AF surgery [21]. Pulmonary vein isolation was achieved through bilateral minithoracotomy incisions and using a dry RF bipolar device. The left atrial appendage was removed with a surgical stapler. Freedom from AF was 91% at 3-month followup. In a subsequent study of 157 patients with up to 4-year followup, they reported a cure rate for paroxysmal AF of 92%, for persistent AF, 85%, and for chronic AF, 75%. Although this technique requires bilateral anterolateral thoracotomies, it has several proposed advantages including (1) lack of cardiopulmonary bypass, (2) exclusion of left appendage, and (3) bilateral autonomic denervation can be performed simultaneously.

Beyer and coworkers performed a multicenter study of 100 patients with atrial fibrillation (39 paroxysmal, 29 persistent, and 32 permanent) using the Wolf technique [46]. The mean operative time was 253 minutes, and the mean length of stay was 6.5 days. Results demonstrated an 86% overall success rate (93% paroxysmal, 96% persistent, and 71% permanent), with 62% discontinuation of antiarrhythmic drugs, and 65% discontinuation of anticoagulation. However, there was a 13% rate of complication (pacemaker implantation, phrenic nerve injury, postoperative hemothorax, and transient ischemic attack) over a mean follow-up time of 14 months.

## 9. Robot-Assisted Minimally Invasive AF Surgery

Loulmet and colleagues first described off-pump robotic endoscopic pulmonary vein isolation with microwave energy (FLEX 10, Boston Scientific Corp., Natick, MA, USA) in a patient in chronic AF in 2004 [47]. Bolotin and coworkers were the first to describe using the da Vinci robot and microwave ablation to treat concomitant AF and mitral valve disease [48]. The same group (Chitwood) reported the results of combined robotic mitral repair and microwave ablation techniques in a series of 16 patients, where 73% were in sinus rhythm 6 months after operation. The microwave ablation probe was introduced through a 4 cm right minithoracotomy to perform epicardial and endocardial pulmonary vein isolation with cardiopulmonary bypass. The da Vinci robot was used to manipulate and position the probe as well as closing the left atrial appendage and repairing the mitral valve [49]. One patient underwent emergent stenting of the left circumflex artery for coronary injury secondary to the microwave ablation. More recently, Cheema and coworkers have described a technique of robotic, endoscopic, and on-pump beating heart left atrial cryoablation; however, further results are awaited [50].

## 10. Totally Endoscopic AF Surgery for Lone AF

Saltman and coworkers first reported a totally endoscopic microwave ablation procedure using 3 access ports bilaterally for paroxysmal AF in 2003 [27]. They later reported a series of 30 patients (50% paroxysmal) with this minimally invasive, purely endoscopic approach [51]. Results suggested a freedom from AF after 12 months of 58% overall, with 70% freedom from AF in patients with paroxysmal disease. There were no deaths in this series; however, minor morbidities were encountered in 16%, ranging from pneumonia to left atrial appendage injury requiring conversion to open thoracotomy.

In 2007, Pruitt and colleagues reported a series of 100 cases of bilateral thoracoscopic microwave ablation; 64% paroxysmal, 11% persistent, and 25% permanent [52]. Unfortunately, their results only achieved a 42% freedom form AF at mean followup of 23 months. Mortality at last followup was 3%. In 9% of patients, the thoracoscopic box pulmonary vein isolation and subsequent electrophysiological intervention failed, and a Cox-maze operation was successfully performed. Yilmaz and coworkers reported completely thoracoscopic pulmonary vein isolation with ganglionic plexus ablation and left atrial appendage amputation using bipolar RF ablation [53, 54]. In their study, AF was paroxysmal in 63%, persistent in 27%, and permanent in 10% of cases. Freedom from AF was obtained in 77% of the patients during a mean followup of 12 months. Edgerton has described a series of patients undergoing bilateral endoscopic ablation with a more comprehensive lesion set, claiming to mimic the Cox-maze III operation [55]. They reported good results in an initial series of 74 patients with 84% and 57% freedom from AF in patients with paroxysmal or longstanding persistent AF at 6-month followup, respectively.

## 11. Concomitant Minimally Invasive AF Surgery and Mitral Valve Surgery

 Minimally invasive techniques within cardiac surgery were first applied to mitral valve surgery [12, 13]; however, because of common surgical exposures, similar minimally invasive options were rapidly developed for AF surgery with the aim of reducing morbidity, postoperative pain, and blood loss [56]. Since mitral valve disease often presents with a high prevalence of AF, there was the natural progression to further develop concomitant minimally invasive therapies to address both disease processes simultaneously. Both endocardial and epicardial approaches have been utilized in addition to multiple energy sources [49, 50].

In 2006, Akpinar and coworkers reported the results of a randomized trial in which 67 patients with permanent AF undergoing minithoracotomy mitral valve surgery with or without AF ablation with unipolar endocardial RF (Cardioblate, Medtronic Corp., Minneapolis, MN, USA) [57]. The pulmonary veins were isolated as pairs, and a lesion was then placed to the mitral valve annulus. Freedom from AF in the ablated group was significantly higher than that in the control group at 1-year followup (94% versus 9%; *P* = 0.0001). Jeanmart and coworkers demonstrated a modified maze with unipolar RF concomitant with minithoracotomy mitral valve surgery resulted in 70% freedom from AF at a mean followup of 17 months [58]. Gillinov and Svensson have developed an approach for creation of biatrial lesion sets for AF ablation with mitral valve surgery through a transseptal exposure and partial upper sternotomy [59].

## 12. Minimally Invasive AF Surgery versus Catheter Abaltion

Catheter ablation is the least invasive procedure for patients with AF refractory to antiarrhythmic medication; however, high recurrence rate for persistent AF remains problematic [33, 34]. In contrast, minimal invasive surgical ablation may be more effective and can include various types of procedures such as pulmonary vein isolation, LAA excision, and ganglia ablation; however, it is still more invasive requiring general anesthesia and a thoracotomy compared to catheter ablation.

Atrial fibrillation catheter ablation versus surgical ablation treatment (FAST) trial is a randomized clinical trial comparing their efficacy and safety [60]. One hundred twenty-four patients with antiarrhythmic drug-refractory atrial fibrillation were randomized to catheter ablation (63 patients) or surgical ablation (61 patients). Catheter ablation consisted of linear atrial pulmonary vein isolation and optional additional lines. Surgical ablation consisted of bipolar RF isolation of the pulmonary veins, ganglionated plexi ablation, and left atrial appendage excision with optional additional lines [60, 61]. Freedom from any left atrial arrhythmia >30 seconds after 12 months was 36.5% for catheter ablation and 65.6% for surgical ablation (*P* = 0.0022). However, the adverse events related with procedural complications was significantly higher for surgical ablation than for catheter ablation during the 12-month followup (34.4% versus 15.9%; *P* = 0.027).

## 13. Hybrid Approach: Minimally Invasive Surgical Ablation with EP Catheter Approach

A hybrid procedure of minimally invasive AF surgery and combined electrophysiology (EP) mapping and ablation has recently been introduced to overcome the shortcomings of each technique individually [62, 63]. In this technique, AF ablation through a minithoracotomy is performed with concomitant EP mapping to confirm conduction block along these lesion sets. Additional surgical and percutaneous ablation is performed to achieve complete block as necessary. Advantages of the hybrid procedure are (1) to confirm conduction block, (2) to maximize efficacy of AF ablation and outcomes, and (3) to minimize potential complications of catheter ablation such as lesion gaps, tamponade, and thrombus formation. This technique does require the expertise and resources of both the surgical and EP teams working together; however, these cases are more time consuming.

This technique was first reported in 2007 as combined procedure with epicardial left atrial ablation and percutaneous endocardial ablation in difficult cases of AF [63]. In 2011, Krul and coworkers reported thoracoscopic video-assisted pulmonary vein antrum isolation, ganglionated plexus ablation, and periprocedural confirmation of ablation lesions for 31 patients (16 paroxysmal AF, 13 persistent AF, and 2 longstanding persistent AF) [64]. Intraoperative EP testing and additional ablation lesions were performed to accomplish complete block. Freedom from AF/atrial flutter/atrial tachycardia without using antiarrhythmic drugs was 86%.

## 14. Left Atrial Appendage Management

Cerebral infarction is the most devastating complication of AF. Blackshear reported that in patients with AF, left atrial thrombi was most commonly found in the LAA in 91% of nonvalvular AF and 57% of valvular AF [65]. Surgical obliteration of the LAA is a concept that is widely accepted during AF surgery; however, the best surgical method has not been determined. Generally, the LAA has been approached from both epicardial and endocardial methods. Epicardial approaches have consisted of (1) excision and oversewing or stapling or (2) proprietary “clip-like” devices. The excision technique confirms removal of the part of the appendage, however, can have risks of hemorrhage and potential thrombus formation in incompletely resected LAA with partial remaining stumps. Epicardial approaches are best suited towards bilateral thoracotomy approaches. Endocardial approaches can be utilized most commonly with a right minithoracotomy approach. Both purse-string and running suture closure techniques (exclusion) have been employed to obliterate the LAA ostium; however, these techniques frequently fail by allowing LAA recanalization [66, 67]. When performing a right minithoracotomy approach (our most common approach), we prefer invaginating the LAA into the left atrium and excising and oversewing of the LAA to confirm elimination. This must be carefully performed to prevent excessive blood losses and prior to concomitant mitral valve repair, as the LAA suture line can be hard to secure safely with a rigid annuloplasty ring in place.

The LAA occlusion study (LAAOS) randomized 77 sternotomy-based patients into occlusion group with suture or stapler and control group [67]. In this study, prophylactic LAA occlusion did not reduce the risk of neurological events. Transesophageal echo (TEE) revealed that successful LAA closure was obtained only in 43% in the suture closure method versus 72% in the stapled group. The negative primary outcome in this study may have been secondary to high failure rate of LAA closure or the small sample size. Kanderian and coworkers reviewed 137 of 2546 patients undergoing surgical LAA closure, who had TEE after surgery by any reason [66]. Fifty-two patients (38%) had the excision, and 85 (62%) underwent exclusion (73 with suture and 12 with a stapler) of the LAA. Although overall successful closure was only 40%, the success rate was significantly different among the groups; 73%, 23%, and 0% in excision, suture exclusion, and stapler exclusion, respectively. LAA thrombus was present in 41% of patients with unsuccessful LAA exclusion versus 0% in the entire excision group. They concluded the LAA excision was the most reliable method. The other interesting finding from both LAAOS and Kanderian's report, the suture exclusion tended to fail due to persistent flow into the LAA, while stapled exclusion group tended to fail due to the presence of a remnant appendage defined as larger than 1 cm [66, 67].

Garcia-Fernandez and coworkers investigated 205 patients undergoing mitral valve surgery and compared 58 patients with LAA ligation and 147 patients without LAA occlusion [68]. They reported that both incomplete LAA occlusion and no LAA occlusion were major risk factors for thromboembolism. Interestingly, they also suggested that incomplete LAA occlusion was more dangerous than no LAA occlusion. In a review by Chatterjee and colleagues, they reported that 7 of 10 studies with different LAA occlusion techniques had positive effect on reducing stroke risk; though the success rate was highly dependent on the closure technique [69]. As a result, they recommended complete closure of the LAA by excision and oversewing to ensure optimal results. The most feared complication of LAA excision remains hemorrhage, particularly in frail, elderly patients with thin and fragile LAA. Pericardial reinforced techniques may be one way to minimize bleeding risks [70].

In terms of drawbacks of LAA obliteration, there are a few reports about the impact of LAA exclusion on left atrial function. Isobe and coworkers developed bilateral appendage preserving maze procedure in 2001 [71]. They concluded that the bilateral appendage preserving maze procedure improved atrial transport and atrial natriuretic peptide secretion without decreasing the effectiveness of maze procedure against AF. Kamohara and coworkers described that LAA exclusion may affect left atrial reservoir function in short- and mid-term periods in animal models [72]. Yamanaka and coworkers reported the influence of LAA exclusion on left atrial function by computed tomography [73]. In their study, LAA preservation contributed to improved transport function, and none of their patients in sinus rhythm after the maze procedure experienced a thromboembolic event.

Management of the left atrial appendage in minimally invasive surgery varies depending on the surgical approaches including bilateral mini-thoracotomy, right mini-thoracotomy, or percutaneous occlusion. Bilateral thoracotomy approaches allow direct access to the left atrial appendage and, henceforth, allows for all conventional techniques of epicardial resection, stapling, or clip devices. The Wolf procedure allows for stapler resection of the LAA through the left thoracotomy [61]. Recently, clip type LAA occlusion devices have been developed for thoracoscopic approach. In 2010, Salzberg et al. reported LAA clip occlusion with 100% success rate [74]. In right minithoracotomy approaches, commonly utilized with concomitant mitral valve surgery, LAA exclusion with purse-string or running sutures has been the most common modality used. However, concerns of high failure rates and early recanalization have tempered these approaches [67]. As a result, invagination of the left atrial appendage into the body of the left atrium, excision and direct suturing, as was performed in the original Cox-maze operation is probably the most efficacious method to achieve complete LAA occlusion via this minimally invasive approach.

Percutaneous transcatheter closure devices implanted in the catherization lab are also currently available. Three transcatheter devices have been developed: the Percutaneous Left Atrial Appendage Transcatheter Occlusion (PLAATO) device (eV3, Plymouth, MN, USA) [75], Watchman device (Atritech Inc., Plymouth, MN, USA) [76], and the Amplatzer Cardiac Plug (ACP) (ASO, AGA Medical/St. Jude Medical, St. Paul, MN, USA) [77]. All three devices are constructed from self-expandable nitinol frame and polymeric membrane. They are delivered percutaneously through a transseptal approach into the left atrium.

The PLAATO device was the first device developed and consists of a self-expandable nitinol cage with a polytetrafluoroethylene membrane [75]. It was studied in 111 patients with contraindications to oral anticoagulation and demonstrated successful implantation rates of 97%. There were six major adverse events, including one death and two strokes during a mean followup of 9.8 months. The authors reported a stroke rate of 2.2%/year compared with the estimated annual stroke rate of 6.3% (using CHADS2 score).

The Watchman device is a parachute-shaped device with fixation barbs for secure implantation within the LAA [76]. The Watchman device is more flexible than the PLAATO device and has less need for oversizing in the LAA orifice. The Watchman Left Atrial Appendage System for Embolic Protection in Patients with Atrial Fibrillation (PROTECT-AF) trial evaluated the device in a prospective, randomized design involving 707 nonvalvular patients with CHADS2 score ≥1 [78]. The patients were randomized to receive either the LAA occlusion device or long-term warfarin therapy. These trial results demonstrated the noninferiority of the Watchman device compared with long-term warfarin therapy group. In the Watchman group, there was a 38% reduction in primary efficacy, 29% in stroke, and 38% in death compared with the long-term warfarin therapy group. However, there was a 77% increase in primary safety events in the Watchman group. Specifically, procedural/device complications occurred in 11% of the Watchman group: procedural stroke 1%, device embolization 1%, significant pericardial effusion 5%, and bleeding 4%. A recent analysis of the nonrandomized Continued Access Protocol registry including 460 subsequent patients after PROTECT-AF study demonstrated a significant improvement in device/procedure-related complications of during the latter half of PROTECT-AF study [78]: procedural stroke 0%, device embolization 0%, significant pericardial effusion 2%, and bleeding 1%. This improved safety was attributed to increased operator experience and device improvements.

The third available option is the Amplatzer Cardiac Plug (ACP) device, which consists of a lobe for fixation in the LAA and a disc for covering the mouth of the LAA like a pacifier. Pilot study results have demonstrated successful device implantation in 96% of patients, with serious complications such as ischemic stroke, device embolization, and pericardial effusion observed in 7% [77]. A randomized trial is underway to assess the ACP device [79].

## 15. Quality of Literature/Future Developments

As the treatment of AF continues to evolve, so do minimally invasive approaches to surgical ablation including the energy sources, surgical approaches, and lesion sets. Current and future therapies must be based upon good evidence to ensure optimal patient outcomes; however, most research in surgical therapies for AF consist of small case series or non-randomized data with follow-up periods of less than 2 years. As lesion sets and energy sources change frequently, there is a dire need to exercise rigorous randomized clinical trial design to assess the efficacy of these surgical techniques. Followup must be complete and employ continuous event monitors rather than most studies which rely upon the inaccuracies of telephone followup and “spot” electrocardiograms. The definition of recurrent AF following an ablation procedure remains controversial (i.e., a defined time period versus clinically relevant outcomes associated with AF) and requires strict definition prior to embarking on a randomized, clinical trial [80]. Considering the high failure rates of left atrial appendage ligation, patients should always be reassured of complete left atrial appendage closure with an imaging modality. Patient followup is essential to proving the true worth of all ablation techniques. Ultimately, any ablation technique will only be efficacious if it reduces symptoms and late complications of stroke, congestive heart failure, and death.

## 16. Summary

Surgical therapies for AF continue to evolve. Patient demand for minimally invasive techniques will exponentially grow. In the traditions of our surgical forefathers, we must continue to innovate and refine less invasive methods that focus on providing the superior results of the Cox-maze procedure with endoscopic approaches that facilitate quicker patient recovery, faster healing, improved cosmetics, and overall lower patient morbidity.
